# Comparison of three different strategies to treat sciatic nerve regeneration: an experimental study

**DOI:** 10.1590/acb370501

**Published:** 2022-08-12

**Authors:** Pedro Henrique Smaniotto, Cristina Pires Camargo, Marcia Saldanha Kubrusly, Rolf Gemperli

**Affiliations:** 1 Plastic Surgeon. Universidade de São Paulo – Medical School – São Paulo (SP), Brazil.; 2 PhD. Universidade de São Paulo – Medical School –Microsurgery and Plastic Surgery Laboratory – São Paulo (SP), Brazil.; 3 PhD. Universidade de São Paulo – Medical School –Transplantation and Liver Surgery – São Paulo (SP), Brazil.; 4 Full Professor. Universidade de São Paulo – Medical School – Plastic Surgeon Division – São Paulo (SP), Brazil.

**Keywords:** Nerve Regeneration, Sciatic Nerve, Jugular Veins, Stem Cells, Microsurgery

## Abstract

**Purpose:**

To compare the effect of vein conduit filled with adipose tissue stem cells (ASC) on peripheral nerve injury regeneration.

**Methods:**

We analyzed 30 male Wistar rats surgically submitted to a 5-mm gap on the sciatic nerve. Then, the animals were divided into three groups: nerve autografting (AG, n=10), autogenous inverted glycerol-conserved vein (VG, n=10), and autogenous inverted glycerol-conserved vein + ASC (VASCG, n=10). The study endpoints were neuromotor functional analysis, gastrocnemius muscle weight, and sciatic nerve graft histomorphometry analysis. In the histologic analysis, we added a control group (naïve nerve).

**Results:**

Regarding functional analysis (Walking tract- score), the findings at week 3 showed a difference between the AG and the VG (-96.6 *vs*. -59.6, p=0.01, respectively) and between the VG and the inverted vein + VASCG (-59.9 *vs*. -88.92, p=0.02). At week 12, this study showed a difference between the AG and the VG (-64.8 *vs*. -47.3, p=0.004, respectively), and also a difference between the VG and the VASCG (-47.3 *vs*. -57.4, p=0.02, respectively). There was no difference in the histomorphometry analysis (nerve diameter, Schwann cells counting). The gastrocnemius muscles on the intervention side were more atrophic when compared to the gastrocnemius muscles on the control side.

**Conclusions:**

Our results suggested better functional recovery in the inverted vein group when compared to control group, and inverted vein + ASC group.

## Introduction

Peripheral nerve injury (PNI) is frequent due to location and fragility of nerves. In the United States, the prevalence of trauma-related PNI varies between 2.8 and 5%^1^,^2^, and treatment is estimated to cost about US$ 150 billion annually^3^.

Autologous nerve grafting is the gold-standard treatment for loss of nerve substance (>2-3 cm gap)^4^,^5^. However, recovery of motor and sensitive functions is incomplete and frequently disabling. Because of neurorrhaphy unsatisfactory results, the peripheral nerve repair treatment requires different wound healing approaches (inflammatory reaction modulation, growth bioreactors, pluripotent stem cells).

Nerve conduits, neural growth factors, and stem cell therapy are new adjuvant therapies for nerve graft surgery.

Several conduits have been analyzed, including the use of conduits of biological origin (BC), such as the inverted vein, which is considered one of the best options for BC^3^,^5^.

Glycerol-preserved veins do not show signs of anatomical demise, which enables their microsurgical use, and renders them less immunogenic^6^, enhance the microenvironment^7^,^8^.

The use of stem cells aims to increase nerve regeneration through cellular differentiation and proliferation in nerve and glial cells. Additionally, the paracrine effect of adipose tissue stem cells (ASC) promotes anti-inflammatory action^9^^-^ 12.

Thus, in the present study, we analyzed the effect of a biological conduit (glycerol-preserved vein) and the association of this conduit with ASC in regeneration of sciatic nerve injury in an animal model.

## Methods

The study was approved by the Ethics Committee for Animal Use of the Universidade de São Paulo Medical School (protocol number 039/15). The study followed ethical principles for animal research according to the National Council of Animal Experimentation (2013).

In this study, 35 isogenic Wistar male rats (*Rattus norvegicus*), at around 8 weeks of age and weighing between 200-250 grams, were kept in a warm controlled environment (24°C), with day/night cycles of 12/12h. Water and food were provided *ad libitum*.

### Harvesting and preparation of jugular veins – vein conduit

Five isogenic rats were used for harvesting adipose tissue and subsequent preparation of adipocyte-derived stem cells.

All animals were anesthetized by the association of ketamine hydrochloride (Ketamin®, Cristalia, Brazil), 100 mg/kg, and xylazine hydrochloride (Rompun®, Bayer, Brazil), 35 mg/kg. They were positioned in horizontal dorsal decubitus, and the right cervical area was shaved. The right external jugular vein was exposed and mobilized. We resected 15 mm of the external jugular vein and ligated the transected ends to prevent hemorrhage. Subsequently, the resected vein specimens were washed with saline solution and preserved in 50% glycerol solution and stored at 4°C for seven days. After this period, each resected vein specimen was placed on an individual 98% glycerol tube for the surgical procedure.

**Figure 1:**
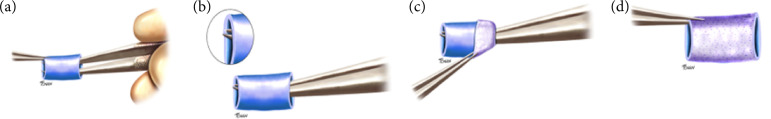
Inversion of the vein graft process. **(a)** With the two tips catheterizing the lumen of the vein, the microsurgical titanium instrument (microsurgical forceps with angulated tips) was introduced all the way to the other end of the tubular structure (vein). **(b)** Clamping of one of the faces of the vein. **(c)** Reversing the vein inside out with the aid of an auxiliary microsurgical forceps with straight tips. **(d)** Result of the graft vein inversion process with the adventitia facing the lumen of the graft, and the endothelium facing the outside.

Immediately before the surgical procedure on the nerve, all resected vein specimens were inverted. To rehydrate veins conduits, they were kept in a 0.9% saline solution for 30 minutes, and then we proceeded with the inversion manipulation, which eventually makes the adventitia face the lumen of the vein conduit, and the endothelium the outside. The inversion was performed manually (Fig. 1).

### Harvesting and preparation of adipocyte-derived stem cells

Five animals were anesthetized by intraperitoneal injection of ketamine hydrochloride (Ketamin®, Cristalia, Brazil), 100 mg/kg, and xylazine hydrochloride (Rompun®, Bayer, Brazil), 35 mg/kg.

One-cm long inguinal incisions were performed bilaterally. The adipose tissue was removed by blunt dissection. Then, the adipose block was reduced to a 1-mm long tissue with a scissor and forceps. The harvested adipose tissue was placed in 25 mL of phosphate buffered saline (PBS) with 1% penicillin G/streptomycin (P/S) added. Trypsin/EDTA was used for enzyme digestion of the material. Then, the material was supplemented with 10% fetal bovine serum (FBS) and 1% P/S antibiotics in Dulbecco’s Modified Eagle Medium (DMEM). Cell supernatant was washed with 2 mL of DMEM twice, and 100 µL of the material was used for cell viability analysis. Cells were maintained in 5% CO_2_ at 37°C until confluency exceeded 80%. Cells were digested with 0.25% trypsin and then frozen at -80°C. The digestion was terminated with DMEM, and cell suspension was centrifuged at 1,800 rpm for 5 minutes and rinsed twice with DMEM. After discarding the supernatant, the pellet was resuspended in 2 mL of DMEM for cell counting in the Neubauer chamber. We used the P4 for this protocol and prepared the concentration of 1 × 10^6^ cells per animal.

### Flow cytometry for adipose tissue stem cells

Flow cytometry was performed using the following antibodies (BD Horizon^TM^ and BD Pharmingen^TM^):

- V450 Hamster Anti-Rat CD29 (0.2 mg/mL), PE Mouse Anti-Rat CD31 (0.2 mg/mL), FITC Mouse Anti-Rat (positive control);
- CD44H (0.5 mg/mL) and APC-CyTM 7 Mouse Anti-Rat (positive control);
- CD45 (0.2 mg/mL); in the well identified as unlabeled, 100 µl of magnetic-activated cell sorting (MACS) buffer (negative control) was added;
- CD 31 (0.2 mg/mL); in the well identified as unlabeled, 100 µL of MACS buffer (negative control) was added.

Sample acquisition was performed in the Fortessa LSR Flow Cytometer using the FACS-Diva^TM^ v 6.13 program (BD Biosciences^TM^). A total of 10,000 events was performed on the FACSCalibur flow cytometer. Cell Quest software (BD Biosciences San Jose, CA, United States of America) was used for data analysis.

The results were biomarkers CD31 and CD29 negative controls, while CD45 and CD44 were positive controls.

### Surgical procedure

Thirty animals were anesthetized with an intraperitoneal injection of ketamine hydrochloride (Ketamin®, Cristalia, Brazil), 100 mg/kg, and xylazine hydrochloride (Rompun®, Bayer, Brazil), 35 mg/kg, and were positioned in ventral decubitus. After shaving the skin of the posterior area of the right hindlimb with an electric razor, all animals were submitted to a 5-mmlong resection of the right sciatic nerve, proximal to its bifurcation at the thigh.

Then, the animals were divided into three groups (10 animals each):

- Autogenous nerve graft group: autograft group (AG);
- Autogenous inverted glycerol-preserved vein graft: inverted vein group (VG);
- Autogenous inverted glycerol-preserved vein graft filled with adipose tissue: inverted vein group + ASC group (VASCG).

All the sutures were performed under microscopic view (Zeiss, United States of America), 4x magnification with 10-0 polyamide suture (Mononylon, Ethicon, United States of America).

**Figure 2:**
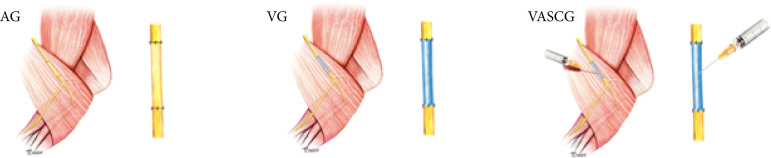
AG: autograft group; VG: inverted vein group; VASCG: inverted vein + ASC group. Methods adopted in the experimental surgeries.

The animals were submitted to an end-to-end suture between the extremities of the vein conduit and the nerve stump, and a group like the previous one, with ASC injected into the vein lumen (inverted vein + ASC group) (Fig. 2).

### Neuromotor functional analysis: walking track test

The neuromotor functional assessment of sciatic nerve regeneration was performed using the walking track (WT) method^13^ preoperatively (immediately before surgery) and postoperatively (three, six, nine, and 12 weeks after surgery).

### Gastrocnemius muscle weight

At the 12th postoperative week and after the WT test, animals were euthanized. Gastrocnemius muscles were collected from the intervention and control sides, right and left hindlimb, respectively.

The posterior compartment of both hindlimbs was exposed, and the gastrocnemius muscles were sectioned at proximal (femoral condyles) and distal (calcaneal) insertions. Afterwards, the muscles (right and left) were weighed on a high precision electric digital scale (Precision – PR-100 110/220 volts, Dourado LTDA).

### Microscopic analysis

We collected the sciatic nerve from operated hindlimbs. Also, we collected a sciatic nerve without any intervention in the contra-lateral hindlimb (n=10) as an additional control group, only in the microscopic analysis. The segment of sciatic nerve collected was 8-mm long to include the microsurgical neurorrhaphy. The nerve segment was divided into five sections:

- Proximal pre-neurorrhaphy: cut I;
- Inter-neurorrhaphy 1: cut II;
- (BC) inter-neurorrhaphy 2: cut III;
- Inter-neurorrhaphy 3: cut IV;
- Post distal neurorrhaphy: cut V.

Specimens were fixed in 4% formalin for 24 h, embedded in paraffin for hematoxylin-eosin (HE) and Manson’s trichrome staining.

### Histomorphometry analysis

By using the histomorphometry technique, we analyzed the following endpoints:

- Number of neuromas;
- Number of effective axons (Schwann cell nuclei);
- Neural bundle diameter (myelination).

The histomorphometry count was performed using the microscope (Nikon, Japan) with the 40x magnification.

### Statistical analysis

For sample size calculation, we chose the significance level of 0.05, study power of 80% and clinical difference between the control and intervention groups of 0.38 with a standard deviation of 0.14. Thus, the estimated sample size was 10 animals per group^14^.

Results were described according to type of variable and its distribution. Numerical variables were tested for normal distribution and range. Parametric variables were described by means and standard deviations.

Inferential analysis was performed for sciatic functional index (SFI) variables, gastrocnemius muscle weight, number of effective axons (Schwann cell nuclei), and neural bundle diameter (myelination). Functional analysis data were compared by periods (three, six, nine, and 12 weeks after surgery). The histomorphometry analysis was performed comparing control nerves (no surgical manipulation) with 12-week post-treatment nerves.

Analysis of variance by rank (Kruskal-Wallis) was used to averiguate the abovementioned comparisons. In case of significance (*p*<0.05), the Dunn’s test was used.

For all data comparisons, we accepted a significance level of 5% and study power of 80%. Statistical analyses were performed using Stata v14 (StataCorp, College Station, TX, United States of America).

## Results

No complications were observed during the study.

### Analysis of neuromotor function: walking track test

The inverted vein group showed better results for the analysis of neuromotor function assessed by the WT test (p=0.009) (Table 1).

**Table 1:** Change in the sciatic function index in the autograft, inverted vein, and inverted vein + ASC groups in relation to pretreatment condition and according to the moment of assessment. Values described as median and IQR.

Groups	Week 3 (median and IQR)	Week 6 (median and IQR)	Week 9 (median and IQR)	Week 12 (median and IQR)
Autograft	-96.6 (-70.25 to -100.19)	-77.04 (-63.51 to -92.53)	-70.28 (-48.53 to -77.75)	-64.8 (-53.9 to -75.1)
Inverted vein	-59.6 (-52.02 to -73.92)	-60.25 (-51.23 to -62.18)	-67.3 (-54.4 to -73.6)	-47.3 (-40.9 to -65.5)
Inverted vein + ASC	-88.92 (-84.08 to -96.4)	-76.6 (-56.6 to -81.5)	-64.8 (-53.9 to -75.8)	-57.4 (-49.9 to -67.4)

ASC: adipose tissue stem cells; IQR: interquartile range.

There were differences among the groups regardless of the time point (p=0.004). There was no difference among the groups at the following moments: pre-treatment, six weeks, nine weeks. At week 3, there was a difference among the groups (p=0.02). The post hoc test (Dunn’s test) showed a difference between the autograft group and inverted vein group (-96.6 *vs*. -59.6, p=0.01, respectively), and between the inverted vein group and inverted vein + ASC group (-59.9 *vs*. -88.92, p=0.02).

At week 12, there was a difference (p=0.02) among the groups. The post hoc test (Dunn’s test) showed a difference between autograft group and the inverted vein group (-64.8 *vs*. -47.3, p=0.004, respectively), and between the inverted vein group and the inverted vein + ASC group (-47.3 *vs*. -57.4, p=0.02, respectively).

### Gastrocnemius muscle weight

The gastrocnemius muscles on the intervention side were more atrophic when compared to the gastrocnemius muscles on the control side. Table 2 shows the weight of the gastrocnemius muscles obtained in each group, as well as their respective contralateral gastrocnemius muscle weight.

**Table 2:** Gastrocnemius muscle weight for each group and respective contralateral sides (control nerve group).

Groups	Gastrocnemius muscle weight (mg) (median, IQR)
Control (n=10)	248 (236 – 279)
Autograft (n=10)	138 (104 – 159)
Inverted vein (n=10)	112 (91 – 146)
Inverted vein + ASC (n=10)	113 (90 – 133)

ASC: adipose tissue stem cells; IQR: interquartile range.

The control nerve group showed a heavier gastrocnemius muscle when compared to the other groups. Comparison among all intervention groups (autograft, inverted vein, and inverted vein + ASC) showed no difference.

### Microscopic analysis

We did not identify any neuroma in all samples.

### Histomorphometry analysis

#### Effective axons

**Figure 3:**
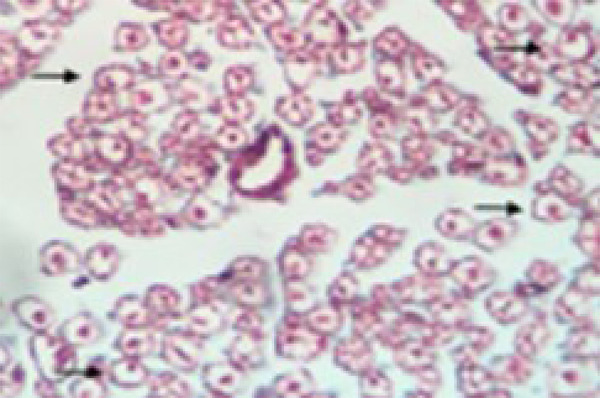
Arrows: effective axons myelinated with Schwann cells (nuclei) in their periphery revealing neuronal regeneration in the vein conduit group. Transverse section with Masson’s trichrome staining under a Nikon E200 microscope (40x).

The comparison among all groups showed differences (p=0.001). The pair-wise comparison showed differences between groups. The control group showed less Schwann cells when compared with the autograft group (p=0.002), inverted vein group (p<0.001), and inverted vein + ASC (p=0.01) (Table 3, Fig. 3).

**Table 3:** Number of effective axons (Schwann cell nuclei) according to treatment group and location of nerve sections. Values described as median – IQR.

Location of nerve section	Control	Autograft(median – IQR)	Inverted vein(median – IQR)	Inverted vein + ASC (median – IQR)
Proximal – pre-graft	45.5 (42 – 51)	145.5 (129 – 212)	376 (323 – 420)	138 (131 – 156)
Proximal – graft	48.5 (45 – 55)	134.5 (121 – 220)	338.5 (317 – 367)	128.5 (121 – 139)
Medial – graft	49.5 (37 – 56)	137 (117 – 207)	321 (207 – 340)	124 (118 – 147)
Distal – graft	48 (41 – 51)	140.5 (122 – 207)	272 (221 – 325)	113 (110 – 131)
Distal post-graft	46.5 (43 – 51)	119.5 (112 – 206)	231 (202 – 320)	114 (105 – 121)

IQR: interquartile range.

We also analyzed nerve bundle diameter after 12 weeks of neural regeneration. Five sections of the injured sciatic nerve were analyzed. This study showed a difference in the diameter of the nerve among groups (p<0.001). The control (control nerve) groups showed similar nerve bundles in all the analyzed sections (p<0.001). Also, there was no difference in the comparison of the distal section in all groups (Table 4).

**Table 4:** Diameter of nerve bundles after 12 weeks of prospective neural regeneration. Histological measurements of slides were performed at five locations (cross-sections) of the right sciatic nerve: proximal pre-graft or neurorrhaphy, proximal – graft, medial – graft, distal – graft, distal – post-graft diameter values in µm are described as median – IQR.

Location (cross-sections)	Nerve diameter (μm)			
	Control (median – IQR)	Autograft (median – IQR)	Inverted vein(median – IQR)	Inverted vein + ASC (median – IQR)
Proximal – pre-graft	0.06 (0.06 – 0.08)	0.07 (0.05 – 0.09)	0.06 (0.06 – 0.07)	0.06 (0.05 – 0.06)
Proximal – graft	0.06 (0.04 – 0.8)	0.07 (0.05 – 0.07)	0.06 (0.06 – 0.08)	0.05 (0.04 – 0.06)
Medial – graft	0.06 (0.04- 0.8)	0.07 (0.05 – 0.08)	0.06 (0.06 – 0.08)	0.05 (0.04 – 0.06)
Distal – graft	0.05 (0.04 – 0.07)	0.05 (0.053– 0.06)	0.04 (0.04 – 0.05)	0.05 (0.04 – 0.05)
Distal – post-graft	0.06 (0.04 – 0.87)	0.04 (0.03 – 0.05)	0.04 (0.06 – 0.06)	0.05 (0.04 – 0.05)

ASC: adipose tissue stem cells; IQR: interquartile range.

## Discussion

Management of PNI is challenging, and, although neurorrhaphy is considered the gold-standard treatment, nerve functional recovery is unsatisfactory. Thus, new strategies are required to improve neuronal regeneration.

Our study showed a better hindlimb functional recovery (WT test) in the inverted vein group when compared to autograft and inverted vein + ASC groups. Nonetheless, there is controversy in the literature regarding this matter. Several studies have revealed the benefit of the association of stem cell injection in absorbable conduits^15^^-^ 17. For instance, Allbright *et al*.^15^ adopted different nerve gap model (2 mm), and the authors used a vein conduit and stem cells to treat PNI with a small gap. As a conclusion of Albright *et al*.^15^, the association of stem cells and vein conduit can improve the microenvironment and the conduit microenvironment to guide nerve fibers during the regeneration process. Moreover, using vein conduit instead of epineural neurorrhaphy makes the surgical procedure straightforward^18^.

Amid several types of conduits, we chose inverted glycerol-preserved isogenous veins. Glycerol treatment eliminates immunogenic proteins and preserves vessel biomechanical characteristics, such as the endurance and the extracellular matrix structure, which is comprised of collagen and laminin^19^. Some authors criticize using veins as conduit due to the potential risk of tube collapsing, because of the presence of vein valves. To correct this issue, we everted the vein inner layer and made the adventitia side face the vessel lumen, whereas the rigid structure of the adventitia would prevent collapsing of the conduit^19^^-^ 22.

According to the macroscopic and histological analysis performed, no neuroma was observed. This may be explained by the minimally traumatic microsurgical technique performed, minimal number of foreign bodies, and immediate intervention after injury occurrence^2^^,^ 4.

Regarding gastrocnemius muscle weight, this study showed the gastrocnemius muscles on the intervention side were more atrophic when compared to the gastrocnemius muscles on the control side.

To perform the histological analysis, we chose Schwann cells, as they are located more centrally and surround each neuron unit. Schwann cells are located around each axon; thus, they do not experience any effect from the preparation. Therefore, we decided to measure the number of Schwann cells (nuclei), enabling us to obtain a more precise count of effective axons. In the histomorphometric analysis, the control group (naïve nerves), the Schwann cells counting, was smaller than the other intervention groups because there was no injury to cause regenerative process.

Regarding sciatic nerve diameter, this study showed a difference in the diameter of the nerve among groups (p <0.001),only the control group showed the same diameter during the study. Although nerve diameter is a surrogate endpoint, the diameter reflected the regeneration process during the study. Eventhough we added ASC the regeneration process was incompleted.

We hypothesized that the ASC and vein conduit group would show better results than the other groups. Surprisingly, the vein conduit group showed a better response in nerve regeneration. Perhaps, the vein conduit concentrated nerve growth factors in the nerve gap and improved nerve regeneration. Maybe, the ASC injection, a mesenchymal cell, did not differentiate in a neuronal cell (ectodermal cells). Keskin *et al*.^23^ consider that the use of vein guide tubes favors nerve regeneration, as it acts as a biological chamber located between the proximal and distal stumps of the PNI. The biochemical composition of the local microenvironment is critical for the regeneration process^7^^,^ 23. At injured nerve stumps, sectioned axons synthesize and secrete cytoplasm fluid containing neurotrophic factors, which remain confined by vein walls, leading to their accumulation at high concentrations in the vessel lumen. We hypothesize that vein conduits guide neuronal regeneration and as a structure to concentrate the interleukin. Therefore, this conduit could improve and potentialize the neuronal healing process^24^.

This study had some limitations. We used a small size sample, which might have affected outcomes. Additionally, we did not use electroneuromyography to evaluate neuromotor function.

## Conclusion

This study suggests that using inverted glycerol-preserved veins showed better functional results when compared to autograft nerves, and inverted vein associated with ASC. Further investigation is required to test this hypothesis.
